# Extending the Palette of Luminescent Primary Thermometers: Yb^3+^/Pr^3+^ Co-Doped Fluoride Phosphate Glasses

**DOI:** 10.1021/acs.chemmater.3c01508

**Published:** 2023-08-02

**Authors:** Fernando E. Maturi, Anuraag Gaddam, Carlos D. S. Brites, Joacilia M. M. Souza, Hellmut Eckert, Sidney J. L. Ribeiro, Luís D. Carlos, Danilo Manzani

**Affiliations:** † Phantom-g, CICECO - Aveiro Institute of Materials, Department of Physics, University of Aveiro, Aveiro 3810-193, Portugal; ‡ Institute of Chemistry, São Paulo State University (UNESP), Araraquara, São Paulo 14800-060, Brazil; § São Carlos Institute of Physics, University of São Paulo, IFSC-USP, São Carlos, São Paulo 13566-590, Brazil; ∥ São Carlos Institute of Chemistry, University of São Paulo, IQSC-USP, São Carlos, São Paulo 13560-970, Brazil

## Abstract

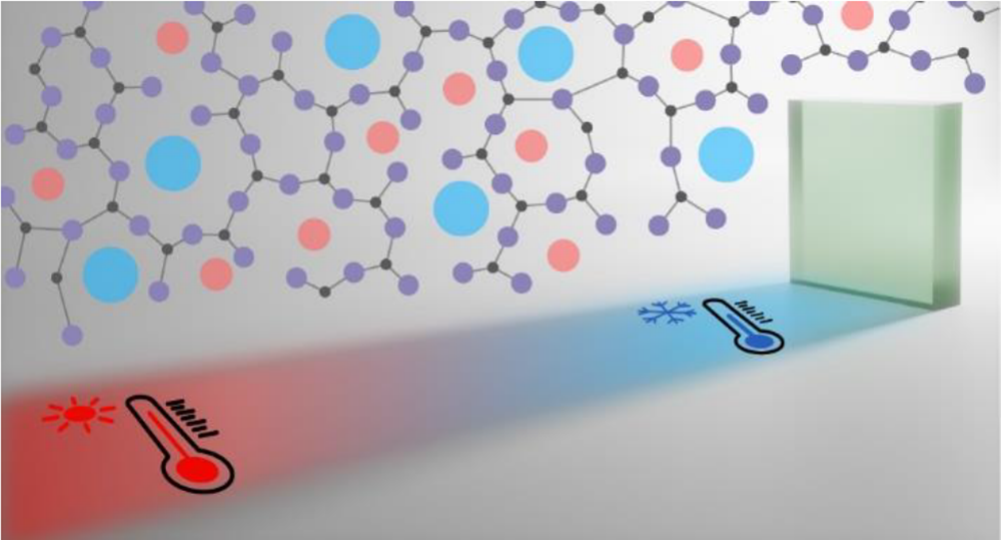

The unique tunable properties of glasses make them versatile materials for developing numerous state-of-the-art optical technologies. To design new optical glasses with tailored properties, an extensive understanding of the intricate correlation between their chemical composition and physical properties is mandatory. By harnessing this knowledge, the full potential of vitreous matrices can be unlocked, driving advancements in the field of optical sensors. We herein demonstrate the feasibility of using fluoride phosphate glasses co-doped with trivalent praseodymium (Pr^3+^) and ytterbium (Yb^3+^) ions for temperature sensing over a broad range of temperatures. These glasses possess high chemical and thermal stability, working as luminescent primary thermometers that rely on the thermally coupled levels of Pr^3+^ that eliminate the need for recurring calibration procedures. The prepared glasses exhibit a relative thermal sensitivity and uncertainty at a temperature of 1.0% K^–1^ and 0.5 K, respectively, making them highly competitive with the existing luminescent thermometers. Our findings highlight that Pr^3+^-containing materials are promising for developing cost-effective and accurate temperature probes, taking advantage of the unique versatility of these vitreous matrices to design the next generation of photonic technologies.

## 1 Introduction

Glasses are essential in the development of optical devices and telecommunication technologies due to their tunable chemical composition that can achieve high ion solubility,^1^ increased transparency,^2^ elevated thermal stability,^3^ and low phonon energies.^4^ With over 400,000 compositions reported to date, glasses play a crucial role in modern civilization.^5^ Among the various types of glasses available, fluoride phosphates have garnered significant interest because they combine the low phonon energies of fluoride glasses with the high chemical, mechanical, and thermal stability of phosphate glasses.^6−9^ Moreover, fluoride phosphate glasses present a high solubility of trivalent lanthanide ions (Ln^3+^), making them suitable for obtaining solid-state lasers,^10,11^ magneto-optical fibers,^12^ and lighting applications.^13,14^ In addition to their unique optical properties, such as narrow absorption and emission bands, long-lived excited states, and high emission quantum yields,^15,16^ Ln^3+^ are promising candidates for luminescence thermometry due to their rich energy level structure.^17^ The temperature-dependent light emission from various luminescent ions has enabled the development of luminescent thermometers for temperature sensing across a wide range of applications.^18−23^

Luminescence thermometry is a versatile, cost-effective, remote, and minimally invasive technique that relies on the temperature dependence of the luminescence of a phosphor with various response parameters, such as the band shape, peak energy or intensity, and excited state lifetimes and rise times.^24^ This technique can afford real-time temperature measurements with high relative thermal sensitivity (*S*_r_ > 1% K^–1^).^25^ Most luminescent thermometers require a calibration procedure to establish the relationship between the photophysical features and the temperature, being classified as secondary thermometers. However, the recording of calibration curves is simply not possible in some applications, such as in biological media,^26^ living animals,^27^ and operating electronic devices.^28^ The strategy used so far in most of these examples was to assume that a calibration curve recorded in one dispersion medium remains valid in other environments, which is a rough estimation that diminishes the accuracy of the luminescent probe due to artifacts arising from spectral distortion.^29,30^

An alternative to the calibration procedure is to perform an absolute determination of the temperature by relying on well-established equations of state, such as those governing ideal gases and blackbody radiation.^31^ These so-called primary thermometers avoid the need for prior temperature calibration, which is a major surplus. Nonetheless, the number of primary luminescent thermometers reported to date remains scarce, with fewer than 20 examples, mainly involving Er^3+^/Yb^3+^ co-doped materials.^32^ Herein, we present, for the first time, a luminescent primary thermometer based on the thermally coupled levels of Pr^3+^. This novel approach is advantageous because (i) the recording of tedious calibration curves is not necessary and (ii) self-heating induced by the excitation source can be neglected. In this work, we thoroughly investigate the preparation route, structural composition, thermal stability, optical properties, and thermometric performance of Pr^3+^/Yb^3+^ co-doped fluoride phosphate glasses used as luminescent primary thermometers.

## 2 Experimental Section

### 2.1 Materials

Lithium carbonate (Li_2_CO_3_, Sigma-Aldrich, 99.9%), ammonium dihydrogen phosphate ((NH_4_)H_2_PO_4_, Alfa Aesar, 98.0%), yttrium fluoride (YF_3_, Strem Chemicals, 99.9%), strontium fluoride (SrF_2_, Sigma-Aldrich, 99.9%), calcium fluoride (CaF_2_, Sigma-Aldrich, 99.9%), praseodymium oxide (Pr_7_O_11_, Lumtec, 99.9%), and ytterbium oxide (Yb_2_O_3_, Lumtec, 99.9%) were used as received from the companies.

### 2.2 Synthesis of Lithium Metaphosphate

Lithium metaphosphate (LiPO_3_) was synthesized through the solid-state reaction between Li_2_CO_3_ and (NH_4_)H_2_PO_4_, where thermal decomposition of the mixture was carried out at 543 K for 12 h in a platinum crucible under an air atmosphere. The temperature was increased, after the decomposition step, at a rate of 10 K min^–1^ up to 1173 K, and the mixture was melted for 30 min before cooling it down to room temperature (298 K). The obtained LiPO_3_ was stored in a glass desiccator for further use.

### 2.3 Preparation of the Glasses

Undoped and Pr^3+^/Yb^3+^ co-doped fluoride phosphate glass samples were obtained by using the conventional melting-quenching method with molar compositions of 50LiPO_3_-20YF_3_-20SrF_2_-10CaF_2_, 98.75[50LiPO_3_-20YF_3_-20SrF_2_-10CaF_2_]:0.25 Pr_7_O_11_/1.00Yb_2_O_3_, and 97.75[50LiPO_3_-20YF_3_-20SrF_2_-10CaF_2_]:0.25 Pr_7_O_11_/2.00Yb_2_O_3_, labeled as PY00, PY14, and PY18, respectively. Further details of the preparation procedure and the chemical composition of the samples are presented in Section S1.1 and Table S1 of the Supporting Information.

### 2.4 Differential Scanning Calorimetry

The differential scanning calorimetry (DSC) curves of the glass samples were registered in a high-temperature calorimeter (DSC 404 F3 Pegasus, Netzsch) to identify the characteristic glass transition temperature (*T*_g_, ± 2 K), the onset temperature of crystallization (*T_x_*, ± 2 K), and thermal stability parameter (Δ*T* = *T_x_* – *T*_g_, ± 4 K) of the obtained glasses. For this, each glass sample (∼15 mg) was placed in an alumina crucible and heated from 298 to 873 K at a heating rate of 10 K min^–1^, under a nitrogen atmosphere (10 mL min^–1^). *T*_g_ and *T_x_* were assigned to the temperatures at which the first derivative of the heat flow *Q* (d*Q*/d*T*) gives the minimum and maximum values around their corresponding peaks, respectively.

### 2.5 Solid-State Nuclear Magnetic Resonance Spectroscopy

Solid-state nuclear magnetic resonance (NMR) studies were performed on an NMR spectrometer (DD2, Agilent) with a field strength of 5.7 T using a 3.2 mm probe with a magic angle spinning (MAS) rate of 8.0 kHz for ^7^Li, and 24.0 kHz for ^19^F and ^31^P MAS-NMR, ^31^P{^19^F} and ^7^Li{^19^F} rotational-echo double-resonance (REDOR)^33^ and constant time REDOR (CT-REDOR),^34^ and ^31^P two-dimensional (2D) J-resolved experiments.^35^ All spectra were analyzed using ssNake^36^ and/or SIMPSON^37^ software. The complete description and experimental conditions of the solid-state NMR experiments are detailed in Section S1.2 of the Supporting Information.

### 2.6 Absorption Spectroscopy

The absorption spectra of the samples in the visible (Vis) and near-infrared (NIR) spectral regions were measured at room temperature (298 K) in a dual-beam spectrometer (Lambda 950, PerkinElmer) over the 400**–**2200 nm range with a resolution of 1.0 nm.

### 2.7 Photoluminescence Spectroscopy and Temperature-Dependent Measurements

The excitation and emission spectra of the Pr^3+^/Yb^3+^ co-doped glass samples in the visible spectral range were acquired in a Fluorolog3 spectrofluorometer (FL3-2T, Horiba), with a TRIAX 320 emission monochromator (fitted with 1200 grooves mm^–1^ grating blazed at 500 nm with a reciprocal linear density of 2.6 nm mm^–1^) coupled to a photomultiplier (R928, Hamamatsu) using the front face acquisition mode and a 450 W Xenon arc lamp as the excitation source. The emission spectra were corrected for detection and optical spectral response of the spectrofluorometer while the excitation spectra were corrected for the spectral distribution of the lamp intensity using a photodiode reference detector. The spectral acquisition in the NIR was performed with the same equipment by using a grating with 600 grooves mm^–1^ blazed at 1200 nm and an H10330A-75 photomultiplier (Hamamatsu) without spectral correction. The temperature was controlled by a helium-closed cycle cryostat coupled to a vacuum system (4 × 10^–4^ Pa) and an autotuning temperature controller (Lakeshore 331, Lakeshore) with a resistance heater. Temperatures were measured with a silicon diode cryogenic sensor (DT-470-SD, Lakeshore) with an accuracy of ± 0.5 K (12–30 K), ± 0.25 K (30–60 K), and ± 0.15 K (60–340 K).

### 2.8 Emission Quantum Yield

The absolute emission quantum yields (*q*) were measured at room temperature (298 K) using a Quantaurus quantum yield measurement system (QY Plus C13534, Hamamatsu) with a 150 W Xenon lamp coupled to a monochromator for wavelength discrimination, an integrating sphere as sample chamber, and two multi-channel analyzers for signal detection in the visible spectral range. The values of *q* for the downshifting emission of Pr^3+^ correspond to the integration over the 450–750 nm spectral range under 443 nm excitation. The reported values present an accuracy of 10%, according to the manufacturer. It was not possible to determine the values of *q* for the NIR emission of Yb^3+^ once it was too faint to be detected by the equipment.

### 2.9 Spectral Deconvolution and Energy Separation Determination

The energy separation Δ*E* between the ^3^P_1_ and ^3^P_0_ thermally coupled levels of Pr^3+^ was estimated from the difference between the barycenters of the emission bands assigned to the ^3^P_1_ → ^3^H_5_ and ^3^P_0_ → ^3^H_5_ transitions in the deconvoluted emission spectra of the glass samples measured at room temperature. The spectral deconvolution and obtained values of Δ*E* are presented in Section S1.3 and Figure S1 in the Supporting Information.

### 2.10 Determination of the Thermometric Parameter

The integrated intensities were obtained by taking the integrated areas in the spectral regions corresponding to the ^3^P_1_ → ^3^H_5_ (*I*_2_, 510–533 nm) and ^3^P_0_ → ^3^H_5_ (*I*_1_, 533–565 nm) transitions of Pr^3+^ from the temperature-dependent emission spectra under excitation at 443 nm. The thermometric parameter Δ was defined as Δ = *I*_2_/*I*_1_. The thermometric performance of Pr^3+^ in the obtained fluoride phosphate glasses was assessed in terms of thermal sensitivity and temperature resolution, as described in Section S1.4 of the Supporting Information.

## 3 Results and Discussion

### 3.1 Thermal Evaluation and Structural NMR Studies

**Figure 1:**
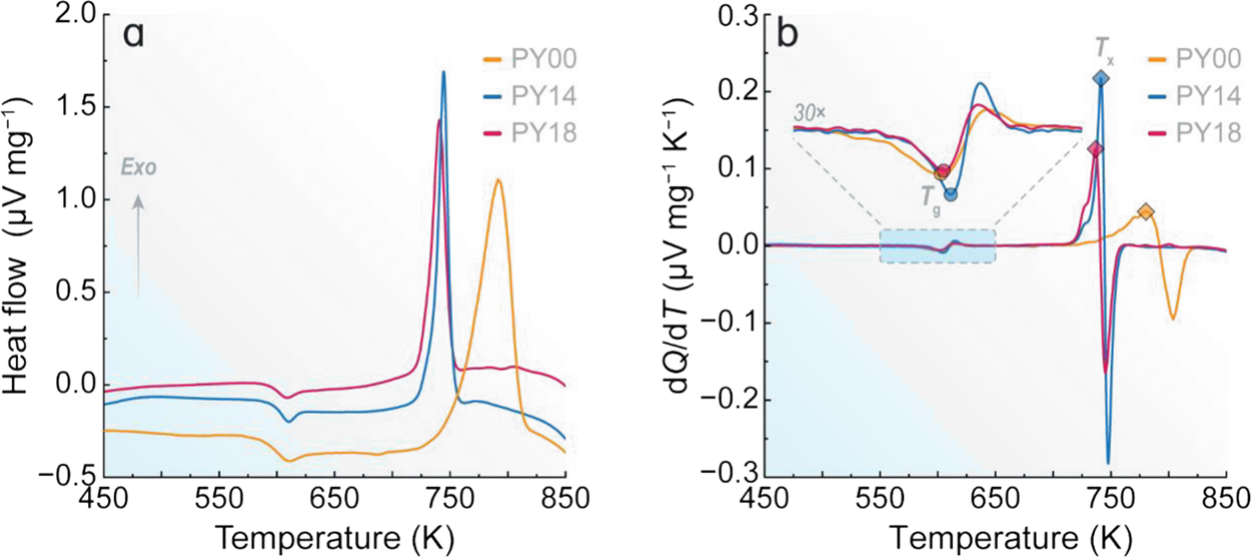
(a) DSC curves and (b) first derivative of the heat flow of the obtained fluoride phosphate samples. The inset in panel b displays the zoomed region of the 550–650 K temperature range. The circles and diamonds indicate the *T*_g_ and *T_x_* temperature values obtained from the onset points of the thermal events, respectively.

Fluoride phosphate glasses were obtained with densities of 3.48, 3.62, and 3.74 g cm^–3^ for the samples PY00, PY14, and PY18, respectively. The increasing density of these glasses follows the increasing Pr^3+^/Yb^3+^ co-doping content, showcasing the influence of the dopant concentration on the physical properties of the glass matrix. The DSC curves of the obtained samples shown in Figure 1 demonstrate that *T*_g_ remains essentially the same regardless of the glasses’ composition, indicating that the addition of Pr^3+^ and Yb^3+^ does not cause significant structural changes in the connectivity of the fluoride phosphate glassy network. Nevertheless, the crystallization peak gets narrower and shifts to lower temperatures when increasing the content of ytterbium oxide, indicating that Yb^3+^ may act as a nucleating agent. Although the thermal stability parameter Δ*T* decreases after co-doping the samples, the obtained values are greater than 100 K, revealing that the obtained glasses present good thermal stability against the devitrification process. The characteristic temperatures of all samples are summarized in Table S2.

**Figure 2:**
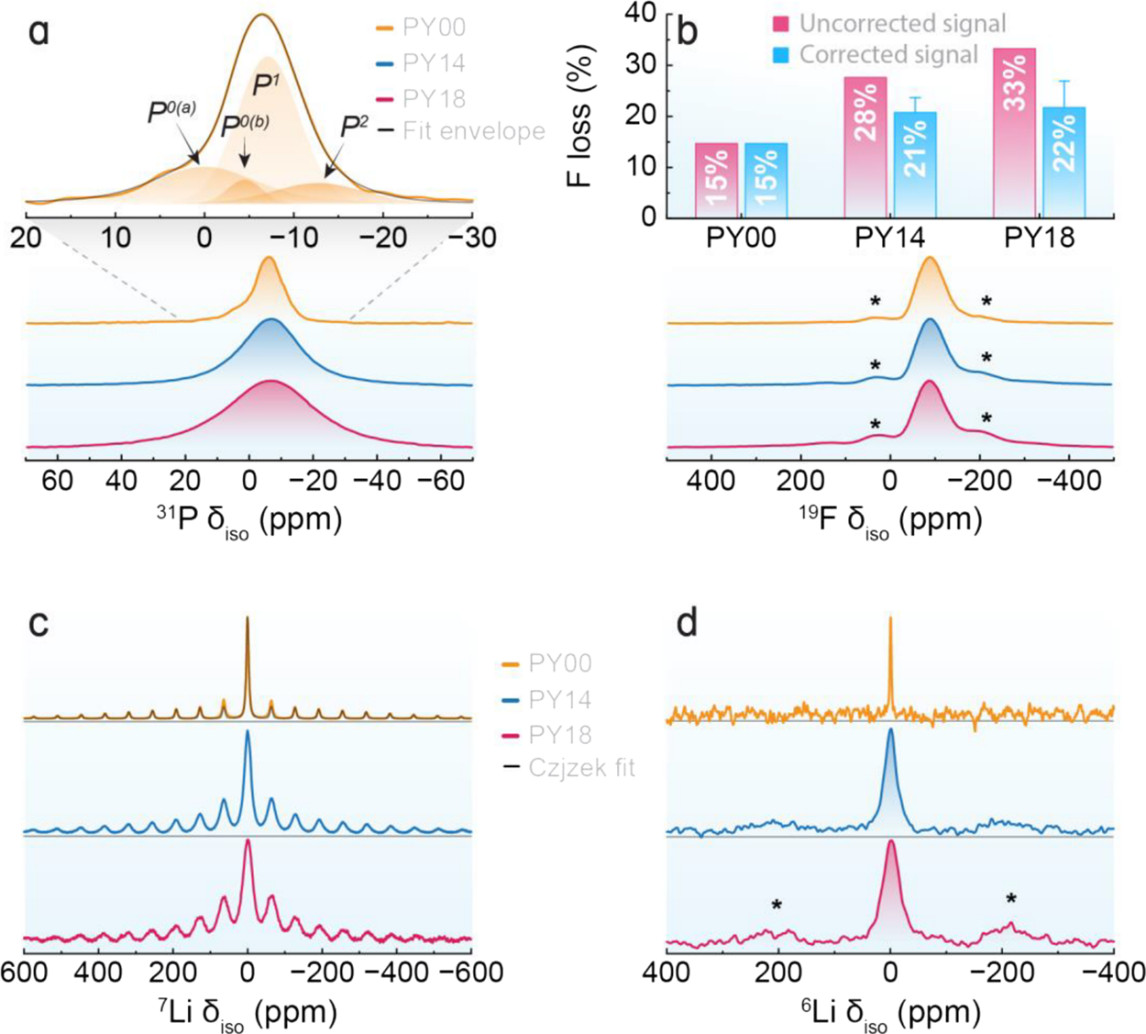
(a) ^31^P MAS-NMR spectra of the undoped (PY00) and co-doped (PY14 and PY18) fluoride phosphate glasses measured at 5.7 T (bottom). Individual lineshape components extracted from the deconvolution analysis of the undoped PY00 sample based on the R-INADEQUATE data (top). (b) ^19^F MAS-NMR spectra of the glasses (bottom). Fluorine loss quantified by ^19^F MAS-NMR (top). The uncorrected signal in pink considers the detection of all F atoms while the corrected signal in blue excludes F atoms coordinated to Yb^3+^/Pr^3+^. Results in blue are mean values from three distinct coordination scenarios with the error bars indicating the deviations between them (see Figure S4). (c) ^7^Li and (d) ^6^Li MAS-NMR spectra of the PY00, PY14, and PY18 samples. The solid black curve in panel c corresponds to the Czjzek fits of the spinning sideband pattern of the undoped glass. Spinning sidebands are indicated by asterisks in b and d.

Figure 2a displays the ^31^P MAS-NMR spectra of the obtained glasses. Sample PY00 presents multiple components which were analyzed in terms of contributions from nonbridged orthophosphate (P^0^, empirical formula PO_4_^3–^), singly bridged pyrophosphate (P^1^, empirical formula P_2_O_7_^4–^), and doubly bridged metaphosphate (P^2^, empirical formula PO_3_^–^) units,^38^ based on additional interaction-selective experiments demonstrated in Section S1.2 of the Supporting Information, where the fitting parameters are listed in Table 1. The major contribution centered at −7.2 ppm arises from P^1^ units (85%), although some contributions from P^0^ and P^2^ units are also evident. This assignment is supported by the 2D J-resolved spectrum of Figure S2, indicating the doublet structure as expected for P^1^ species having P–O–P linkages, with an estimated ^2^*J* coupling constant of 20 Hz. The identity of the P^1^ and the P^2^ units having P–O–P linkages was further confirmed by the double-quantum filtered signal displayed in Figure S3, showing the NMR signal of only those P species that are involved in P–O–P linkages. We note that the P^1^ units identified in the double-quantum filtration experiment show their peak maximum (δ_max_) at −8.7 ppm, whereas δ_max_ in the single-pulse spectrum appears at −6.8 ppm (Table 1). This ∼2.0 ppm difference suggests that another P^0^ contribution may be overlapping the P^1^ signal, as also suggested by a more detailed inspection of the contour plot in the J-resolved MAS-NMR spectrum.

**Table 1:** Fitting Parameters of the Obtained ^31^P NMR Data

sample	units	^31^P R-INADEQUATE				^31^P MAS-NMR			
		δ_max_ (ppm)	δ_iso_ (ppm)	FWHM (ppm)	area (%)	δ_max_ (ppm)	δ_iso_ (ppm)	FWHM (ppm)	area (%)
PY00	P^0(a)^	–8.7				–6.8	–0.1	31	28
	P^0(b)^						–4.5	11	5
	P^1^		–7.2	22	85		–7.1	19	55
	P^2^		–13.6	28	15		–12.7	28	12
PY14						–7.1	–6.6	45	100
PY18						–7.2	–6.5	69	100

Figure 2a also shows the simulation of the ^31^P MAS-NMR spectrum, constrained by the line shape parameters of the P^1^ species via the R-INADEQUATE line shape fit. The possibility for this additional P^0^ signal arising from an FPO_3_^2–^ unit was tested by analyzing the Fourier transforms of ^31^P{^19^F} REDOR difference signals at short dipolar mixing times (Figure S5). As an FPO_3_^2–^ unit is expected to show rapid dephasing in such an experiment, a chemical shift difference between the remaining signal and the difference signal would be expected. This, however, was not observed experimentally. The ^31^P{^19^F} REDOR curve (Figure S6) also gives no evidence of a fraction of rapidly dephasing phosphate species. Still, we cannot exclude the possibility that the minor feature near −4.5 ppm (labeled P^0(b)^ in Figure 2a) was not detectable by ^31^P{^19^F} REDOR owing to sensitivity limitations. Samples PY14 and PY18 show poorly resolved spectra in Figure 2a due to broadening by interactions with paramagnetic Pr^3+^ and Yb^3+^, precluding a detailed deconvolution analysis.

The ^19^F MAS-NMR line shape (Figure 2b) of the undoped sample is slightly asymmetric with the center of gravity (δ_CG_) at −96.1 ppm, whereas δ_max_ is observed near −89.2 ppm, as displayed in Table 2. Considering that the ^19^F chemical shift values for LiF, CaF_2_, SrF_2_, and YF_3_ are −204, −108, −87, and −62 ppm, respectively, it is possible to attribute the observed signal to fluorine (F) in a mixed-metal environment. These results further indicate that the resonance signals of F species not directly coordinated to Yb^3+^ or Pr^3+^ are little affected by paramagnetic broadening. The observed spectra do not show evidence of P-bonded F species, which would give rise to a peak in the vicinity of −70 ppm. Thus, these samples are better described as fluoride phosphate rather than fluorophosphate glasses.^7^

**Table 2:** Fitting Parameters of the Obtained ^19^F, ^7^Li, and ^6^Li NMR Data

sample	^19^F MAS-NMR			^7^Li MAS-NMR			^6^Li MAS-NMR	
	δ_CG_ (ppm)	FWHM (ppm)	δ_max_ (ppm)	δ_iso_ (ppm)	FWHM (ppm)	*C*_Q_ (kHz)	δ_iso_ (ppm)	FWHM (ppm)
PY00	–96.1	76.0	–89.2	0.23	6.0	110	–0.65	1.8
PY14	–96.9	79.0	–88.6	–0.10	21.0		–0.87	22.8
PY18	–98.5	84.0	–87.9	–0.01	32.0		–1.12	30.8

Fluoride phosphate glasses are known to experience F volatilization during synthesis. Hence, the quantitative nature of NMR was used to estimate the amount of F loss by employing ^19^F MAS-NMR. By analyzing the detected ^19^F signal, a significantly higher apparent loss was observed in the paramagnetically co-doped glasses compared to the undoped glass (Figure 2b, top panel), with this loss increasing with the Yb^3+^ content. It is worth noting that once all these glasses were prepared under identical conditions, the fluorine species directly bonded to the paramagnetic dopants cannot be detected due to the excessive paramagnetic broadening of the resonance signal. Although considering 8-fold coordination is a reasonable approximation for estimating the minimum signal loss due to lanthanide ions embedded within a crystalline fluoride structure,^39^ previous diffraction studies from Hoppe et al. have shown that the average coordination numbers of Pr^3+^ and Yb^3+^ in metaphosphate glasses are 7.0 and 6.5, respectively.^40^ Assuming that these average coordination numbers are similar in fluoride phosphate glasses, the signal loss resulting from paramagnetic interactions can be estimated in the case of dominant Pr–F and Yb–F bonding.

Upon correcting for this additional contribution to the signal loss, the estimated evaporation loss is more consistent with that observed in the undoped sample, yielding 15, 20, and 21% F losses for PY00, PY14, and PY18, respectively (Figure S4). It is important to note that these calculations represent the estimation of the average F loss caused by evaporation, where higher evaporation losses would occur if a significant amount of phosphate ions were present in the first coordination sphere of the lanthanide ions. For instance, assuming half of the ligands to be phosphate and the other half fluoride ions, the maximum corrected evaporation losses would give 24 and 27% for PY14 and PY18, respectively. Therefore, Figure 2b displays the F evaporation loss, which presents the uncorrected values and the corrected mean values from calculations considering the coordination number of Pr^3+^/Yb^3+^ in crystalline fluoride (8.00/8.00), vitreous fluoride-only (7.00/6.50), and mixed vitreous fluoride phosphate (3.50/3.25) environments. Nevertheless, the enhanced signal loss observed in the co-doped glasses compared to the undoped sample indirectly indicates significant Pr/Yb–F bonding in the co-doped samples.

Figure 2c,d displays the ^7^Li and ^6^Li MAS-NMR spectra of the fluoride phosphate glasses, respectively, stressing the paramagnetic broadening effects caused by Pr^3+^ and Yb^3+^ doping. For the undoped PY00 sample, the spinning sideband manifold observed arises from the effect of MAS on the *m* = ± 1/2 ↔ *m* = ± 3/2 satellite transitions, which are inhomogeneously broadened by the quadrupolar interactions. Furthermore, a comparison of the spinning sideband patterns on the doped and the undoped samples suggests that paramagnetic interactions have an additional effect on the observed spinning sideband intensity distribution. For the undoped sample, the satellite transitions in the ^7^Li MAS-NMR spectra were fitted with the Czjzek model using the ssNake software (refer to Table 2 for the fitting parameters).^36,41^

As the chemical shift dispersion in ^6/7^Li NMR is extremely limited, further spectral editing and characterization were done exploiting the ^6^Li–^31^P dipole–dipole interactions using a ^6^Li{^31^P} REDOR experiment. In this case, the less abundant isotope ^6^Li must be chosen as the observed nucleus because the resonance frequencies of ^7^Li and ^31^P are too close to enable the necessary double-tuning of the NMR probe. Figure S7 compares the ^6^Li{^31^P} REDOR data of PY00 and LiPO_3_ glass, where the uncorrected second moments extracted from the data by using eq S1 are 0.69 and 0.65 Mrad^2^ s^–2^, respectively, being considered identical within the experimental uncertainty limits of ± 10%. The theoretically calculated *M*_2(Li–P)_ for crystalline LiPO_3_ is 2.69 Mrad^2^ s^–2^, based on eq S2. In addition, an analysis of molecular dynamics simulation output for glassy LiPO_3_ resulted in *M*_2(Li–P)_ = 1.97 Mrad^2^ s^–2^, and the corresponding parabola predictions are included in Figure S7. The substantial deviation of the experimental REDOR data from the latter simulation suggest a calibration factor *f* = 0.33. Most likely, this relatively small value arises from the large ^31^P chemical shift anisotropy interfering with the dipolar recoupling efficiency.

Regardless of this issue, the most surprising result of Figure S7 is the close correspondence of the two experimental values for LiPO_3_ glass and PY00, despite the considerable dilution of phosphate content in the latter glass. It suggests that the lithium ions exercise a strong preference for being bound to phosphate rather than to fluoride ions in this mixed anion glass. Complementary information is available from ^31^P spin echo decay data assessing the strength of the homonuclear ^31^P–^31^P magnetic dipole–dipole interactions (Figure S8). The *M*_2(P–P)_ values extracted from these data are much larger than those expected from a random distribution of ^31^P nuclei in space, as estimated from a Monte Carlo simulation. This is, of course, understandable, as the presence of a considerable fraction of P–O–P linkages brings the P atoms into proximity much more closely than would be expected for a random distribution of P atoms in space. Also, *M*_2(P–P)_ is significantly larger in LiPO_3_ glass than in PY00 glass, because it features two P–O–P linkages per P atom whereas the NMR results show that there is less than one P–O–P linkage on average in the latter case.

Altogether, these NMR results show that alloying LiPO_3_ glass with alkaline-earth and yttrium fluorides produces significant network modification. A substantial fraction of P–O–P linkages is broken leading to the depolymerization of a P^2^-dominated structure into one that is dominated by P^1^ units. The process can be initially visualized as:
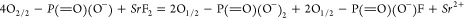
1which may be followed by partial O ↔ F exchange in the melting atmosphere. The latter process is experienced as fluorine loss and may be responsible for our inability to observe specific structural features indicating P–F bonding in the obtained glasses. The ^6^Li{^31^P} REDOR experiment suggests a clear preference for Li-phosphate over Li-fluoride interaction. The formation of P–F bonds appears to be largely suppressed in these samples.

### 3.2 Optical Characterization

**Figure 3:**
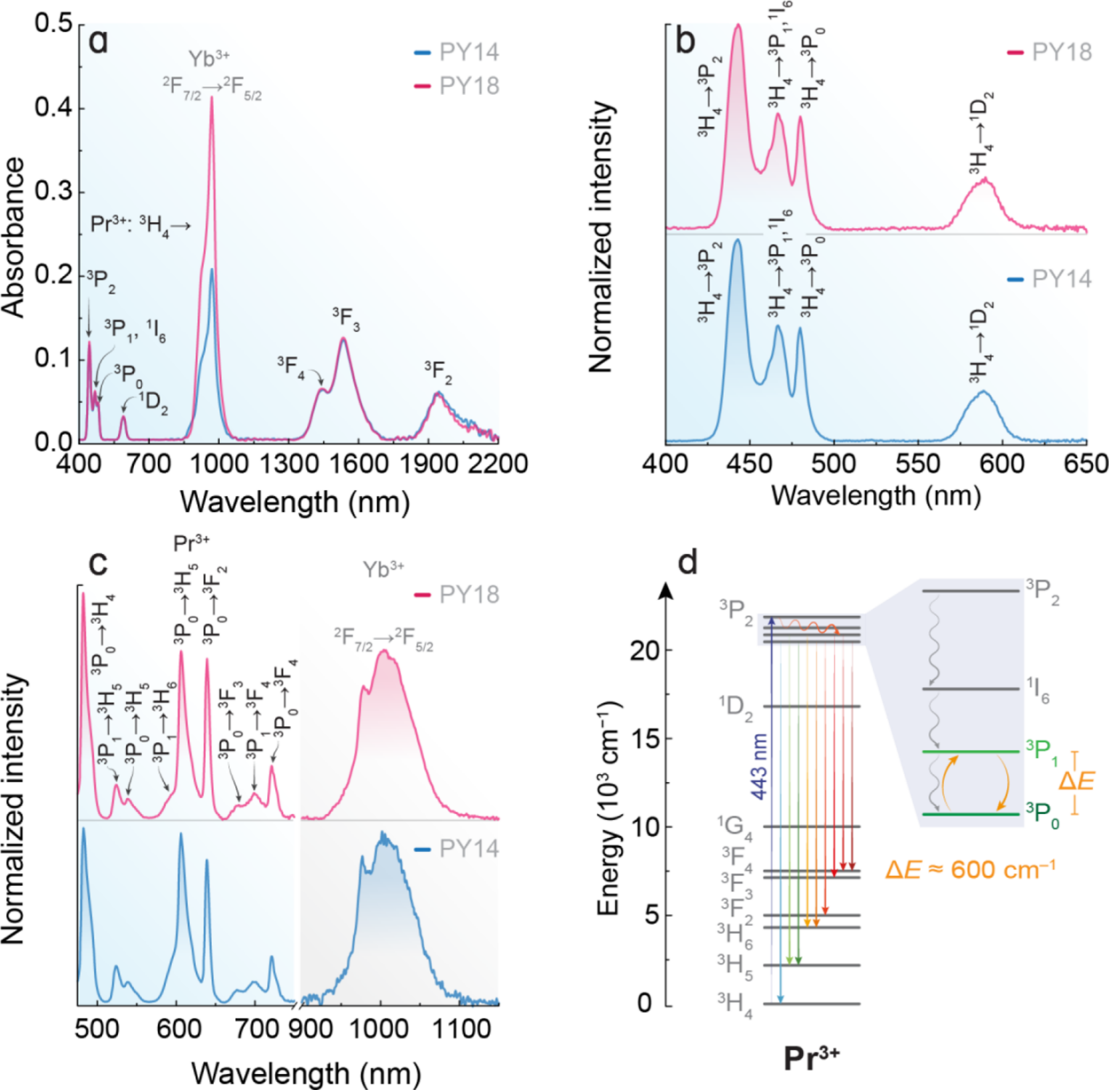
(a) Vis–NIR absorption spectra of the fluoride phosphate glass samples. The absorptions of Pr^3+^ arise from the ^3^H_4_ ground state to different excited states while Yb^3+^ exhibits the ^2^F_7/2_ → ^2^F_5/2_ transition. The absorption spectrum of the sample PY00 is not shown because it is optically inactive due to the lack of Yb^3+^ and Pr^3+^ in its composition. (b) Excitation spectra monitoring the emission at 606 nm and (c) emission spectra in the Vis (Pr^3+^, left) and NIR (Yb^3+^, right) spectral range under excitation at 443 nm of the samples PY14 and PY18 measured at room temperature. (d) Energy level diagram of Pr^3+^ depicting the downshifting emission in the Vis spectral range, underscoring the thermally coupled levels.

The absorption spectra of the colorless undoped and greenish Pr^3+^/Yb^3+^ co-doped fluoride phosphate glass samples are shown in Figure 3a. The Pr^3+^ transitions from the ground state (^3^H_4_) to the ^3^P_2_ (443 nm), ^3^P_1_ and ^1^I_6_ (469 nm), ^3^P_0_ (481 nm), and ^1^D_2_ (588 nm) upper energy levels are observed in the Vis spectral region. In the NIR spectralrange, the absorption bands peaking at 1440, 1533, and 1941 nm are related to transitions from the ^3^H_4_ ground state to the ^3^F_4_, ^3^F_3_, and ^3^F_2_ excited states of Pr^3+^, respectively. The absorption band observed at 974 nm is assigned to the transition of Yb^3+^ from the ground state ^2^F_7/2_ to the excited state ^2^F_5/2_. It is worth pointing out that all the transitions of Pr^3+^ present similar absorbance values because the content of Pr_7_O_11_ is the same in both samples while the absorbance of the ^2^F_7/2_ → ^2^F_5/2_ transition of Yb^3+^ at 974 nm is greater for PY18 once it presents twice the content of Yb_2_O_3_ compared to PY14.

The room-temperature excitation spectra of the Pr^3+^/Yb^3+^ co-doped glasses were recorded by monitoring the emission of the ^3^P_0_ → ^3^H_6_ transition of Pr^3+^ at 606 nm (Figure 3b), displaying the characteristic absorptions of Pr^3+^ centered at 443 nm (^3^H_4_ → ^3^P_2_), 469 nm (^3^H_4_ → ^3^P_1_,^1^I_6_), 481 nm (^3^H_4_ → ^3^P_0_), and 588 nm (^3^H_4_ → ^1^D_2_), in good agreement with the absorption spectra of the samples presented in Figure 3a. Under excitation at 443 nm, both co-doped samples present the typical multicolor emission of Pr^3+^ between the blue and red spectral range (Figure 3c). The emission bands within this spectral region arise from the ^3^P_0_ → ^3^H_4_ (482 nm), ^3^P_1_ → ^3^H_5_ (524 nm), ^3^P_0_ → ^3^H_5_ (539 nm), ^3^P_1_ → ^3^H_6_ (589 nm), ^3^P_0_ → ^3^H_6_ (606 nm), ^3^P_0_ → ^3^F_2_ (639 nm), ^3^P_1_ → ^3^F_3_ (677 nm), ^3^P_1_ → ^3^F_4_ (701 nm), and ^3^P_0_ → ^3^F_4_ (721 nm) transitions of Pr^3+^ in a downshifting emission process.^42^

Additionally, the excitation of PY14 and PY18 at 443 nm also gives rise to a broad emission band peaking at 979 and 1006 nm, with the characteristic emission profile of the ^2^F_5/2_ → ^2^F_7/2_ transition of Yb^3+^ in the NIR spectral range (Figure 3c).^43^ Under 443 nm excitation, the ^3^P_*J*_ manifold and ^1^I_6_ emitting levels of Pr^3+^ may undergo nonradiative decay to the ^1^G_4_ energy level, which is resonant with the ^2^F_5/2_ level of Yb^3+^, resulting in the Yb^3+^ NIR emission around 1000 nm, due to a Pr^3+^-to-Yb^3+^ energy transfer process.^44^ By comparing the absorption and excitation spectra of the PY14 and PY18 glasses (Figure S9), it is possible to observe that the relative absorption strengths of the ^3^H_4_ → ^3^P_2–0_,^1^I_6_ and ^3^H_4_ → ^1^D_2_ transitions (i.e., the ratio between the integrated intensities from the ^3^H_4_ → ^3^P_2–0_,^1^I_6_ and ^3^H_4_ → ^1^D_2_ absorption bands) stay nearly unchanged (Table S3), indicating that the Yb^3+^ NIR emission upon Pr^3+^ excitation is a downshifting mechanism rather than a downconversion emission.^45^ This is further supported by the fact that the absolute emission quantum yield in the NIR cannot be measured under 443 nm excitation, as observed in Table S4.

Glasses containing Pr^3+^/Yb^3+^ are also known for displaying upconversion light emission due to a Yb^3+^-to-Pr^3+^ energy transfer process, where the emission of Pr^3+^ in the Vis spectral range is induced by NIR excitation of the ^2^F_5/2_ level of Yb^3+^.^46,47^ Although upconversion was observed for the PY14 and PY18 co-doped glass samples, it required a high laser excitation power density (> 100 W cm^–2^), with the laser-induced heating resulting in a local temperature increment which was high enough to break the samples. Nevertheless, measurements were performed by using the ground samples prepared for the solid-state MAS-NMR studies, where their upconversion emission spectra under 980 nm laser excitation are presented in Figure S10, displaying the characteristic emission bands of Pr^3+^ in the visible spectral range.

### 3.3 Primary Pr^3+^-Based Luminescence Thermometry

When the energy separation between two emitting levels is sufficiently small (200 to 2000 cm^–1^),^25^ they are considered thermally coupled because they exhibit a thermally induced population distribution between them, following the Boltzmann statistics.^48^ By taking the ratio between the integrated intensity of the emission bands arising from these levels (*I*_1_ and *I*_2_, namely, the integrated emission intensities of the emission bands arising from the lower and upper energetic emitting levels, respectively), a thermometric parameter Δ can be obtained, translating the temperature-induced changes into a luminescence intensity ratio:
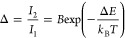
2where Δ*E* is the energy separation between the thermally-coupled levels, *k*_B_ is the Boltzmann constant, *T* is the absolute temperature, and *B* is a pre-exponential factor given by the ratio of the product of degeneracy and total spontaneous emission of both excited states to the ground state.^48^

Most of the studies on luminescent thermometers based on the Boltzmann statistics are calibrated by performing a fitting procedure that enables the estimation of Δ*E* and *B* through eq 2, from the slope and intercept of the ln(Δ) against 1/*T* plot, respectively.^17^ However, this introduces artifacts in temperature determination, mainly when emission spectra are measured outside the luminescent thermometer’s optimal temperature range^49^ or when intruding emission bands (unrelated to the thermally coupled levels) coexist in the same spectral range of *I*_1_ and *I*_2_.^50^ Moreover, once this approach determines the values of Δ*E* and *B* in a temperature-dependent fitting procedure as in secondary thermometers, it is challenging to perform accurate measurements of the temperature outside the calibration range.

To circumvent these issues, Balabhadra et al. have reported a straightforward procedure that can be used to avoid the recording of calibration curves when dealing with the Boltzmann-based luminescent thermometers discussed here.^51^ This methodology consists in determining the parameter Δ_0_, which is the thermometric parameter Δ (i.e., luminescence intensity ratio from two thermally coupled levels) obtained at room temperature (*T*_0_):
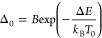
3

This strategy is ingenious because it paves the way for measuring the absolute temperature *T* by taking the ratio Δ/Δ_0_ from eqs 2 and 3:
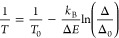
4where *T*_0_ can be easily measured with a temperature sensor, Δ_0_ corresponds to the luminescence intensity ratio from the emission spectra recorded at *T*_0_, and the experimental values of Δ are obtained from the emission spectra recorded at different temperatures. Therefore, it is possible to predict *T* directly from the thermometric parameter Δ by using eq 4. Because Δ_0_ and *T*_0_ correspond to experimentally measured values that are obtained apart from any calibration or fitting procedures, they play the role of normalization factors rather than a calibration process. In addition, Δ*E* is calculated in a nontemperature-dependent way, where this Boltzmann-derived thermometric approach is based on a well-defined equation of state that does not rely on unknown or significantly temperature-dependent values, matching the definition of a primary thermometer.^31^

Even though this methodology precludes the calculation of the pre-exponential factor *B* and can be virtually applied to any Ln^3+^ displaying thermally coupled levels, it has been predominantly employed for primary temperature sensing using the upconverting emission from Yb^3+^/Er^3+^ co-doped materials. The major drawback of this preference is the estimation of Δ_0_ from the intercept of a straight line fitted to the power dependence of Δ, which may introduce errors in the determination of the temperature. In addition, the temperature readouts are affected by local temperature increases induced by the laser excitation required in upconverting approaches, further compromising the accuracy of the temperature measurements.^51^

The use of Pr^3+^ is advantageous in this context because, besides presenting thermally coupled energy levels (^3^P_1_ and ^3^P_0_, Δ*E* ≈ 600 cm^–1^),^52,53^ its downshifting emission can be achieved by using a Xe lamp as the excitation source, where laser-induced heating is absent during the spectral acquisition and Δ_0_ can be determined by simply measuring the emission spectrum at room temperature. The only fitting parameter required to perform primary temperature sensing by applying eq 4 to Pr^3+^ is Δ*E*, which can be easily calculated by deconvoluting the emission spectra measured at room temperature (see Experimental Section). Interestingly, Δ*E* and Δ can be calculated by using the emission bands of Pr^3+^ arising in the 510–565 nm range (^3^P_1_ → ^3^H_5_ and ^3^P_0_ → ^3^H_5_) or the 575–632 nm range (^3^P_1_ → ^3^H_6_ and ^3^P_0_ → ^3^H_6_). Although the latter requires spectral deconvolution to account for the overlapping emission band from the ^3^P_0_ → ^3^F_2_ transition peaking at 639 nm, Pr^3+^ offers two distinct pathways for assessing the temperature-dependent population distribution between the ^3^P_1_ and ^3^P_0_ emitting levels. This allows using the Pr^3+^/Yb^3+^ co-doped fluoride phosphate glasses as primary thermometers based on eq 4 without the need for external calibration,^31^ showcasing the greater advantage of choosing Pr^3+^ over Er^3+^-based materials to perform luminescent primary temperature sensing.

**Figure 4:**
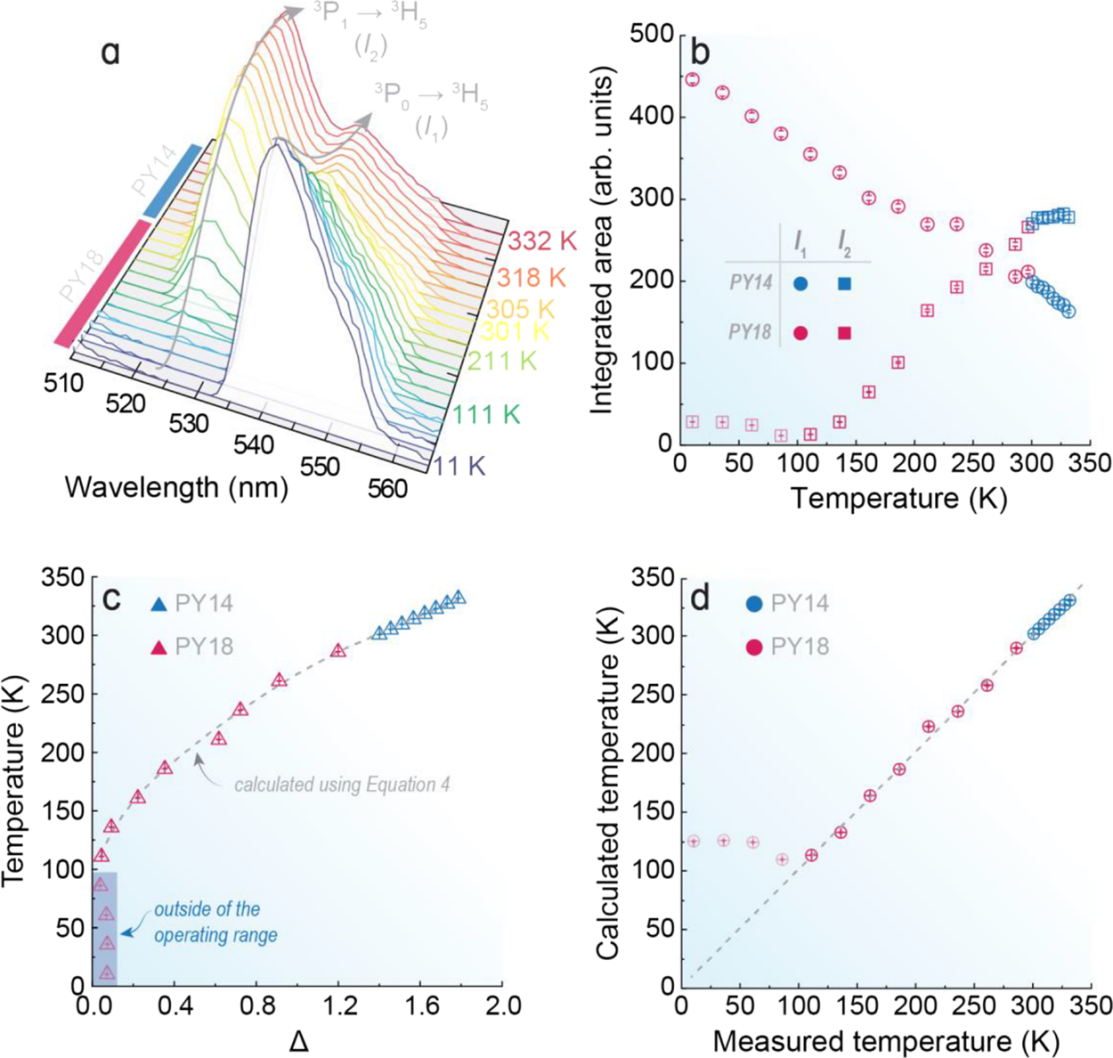
(a) Temperature-dependent emission spectra of the PY14 and PY18 samples under 443 nm excitation, highlighting the emission bands arising from the thermally coupled levels of Pr^3+^. The emission spectra in the complete spectral range recorded are presented in Figure S11. Temperature dependence of (b) integrated emission intensities *I*_1_ and *I*_2_ and (c) corresponding Δ values. The dashed line in c displays the temperature values calculated through eq 4 and their corresponding uncertainties while symbols are the temperature values measured with the reference silicon diode cryogenic temperature sensor. The shadowed area delimits the temperature range where Boltzmann statistics fail. (d) Correlation between the measured with the reference silicon diode cryogenic temperature sensor (*x*-axis) and calculated from eq 4 (*y*-axis) temperature values. The dashed line corresponds to *x* = *y*.

Therefore, by replacing the values of Δ*E*, *T*_0_, Δ_0_, and the experimental values of Δ obtained from the temperature-dependent emission spectra of the obtained samples in eq 4, an excellent agreement between the calculated and measured temperatures was achieved, as observed in Figure 4a. This indicates that the proposed methodology is valid for predicting the absolute temperature, without the need for previously recording a calibration curve. The values of Δ*E*, *T*_0_, and Δ_0_ obtained for PY14 and PY18 are listed in Table S6. Although there is a slight difference in the values of Δ*E* calculated for PY14 and PY18, the integrated intensities of the emission bands related to the thermally coupled levels of Pr^3+^ show the same behavior for the temperature evolution in both samples (Figure 4b). Furthermore, the temperatures predicted by both samples follow the same trend at distinct temperature ranges (Figure 4c,d), indicating that the intrinsic thermal sensing ability of Pr^3+^ is not affected by the chemical environment of the fluoride phosphate glass matrix.

The optimal thermal response of the thermally coupled levels occurs within a specific temperature range that depends on the energy separation Δ*E* between them , encompassing the optimal operating temperature range where the Boltzmann statistics are still valid.^48^ Taking into account that Pr^3+^ presents a Δ*E* of approximately 600 cm^–1^, 253–432 K is the best temperature operating range for the obtained fluoride phosphate glasses co-doped with Yb^3+^/Pr^3+^. Notably, the intensity of the ^3^P_1_ → ^3^H_5_ transition in sample PY14 is comparable to the noise level for temperatures below 111 K, being *I*_2_ near zero (Figure 4a,b). This is because at these temperatures, the nonradiative rates are comparable to the radiative rates, and there is not enough thermal energy to allow the population distribution between the ^3^P_0_ and ^3^P_1_ thermally coupled levels of Pr^3+^. Consequently, only the lower ^3^P_0_ emitting level is populated.^48^ This means that, from an experimental point of view, the primary thermometric approach can be employed in a broader range of temperatures, working well for Pr^3+^ operating above 111 K. Nevertheless, the thermometric performance was herein evaluated at room temperature *T*_0_ to avoid misinterpretations of the thermal response of the ^3^P_1_ and ^3^P_0_ levels of Pr^3+^.

The thermometric performance of Pr^3+^ working as a primary thermometer in the obtained fluoride phosphate glasses was evaluated by using the relative thermal sensitivity (*S*_r_) and uncertainty in temperature (δ*T*), which are crucial figures of merit used to compare the performance of different luminescent thermometers (see Section S1.4 of the Supporting Information for further information).^18^ The *S*_r_ values obtained for both PY14 and PY18 are ∼1.0% K^–1^ at *T*_0_ (Table S6), consistent with previously reported *S*_r_ values for Pr^3+^.^53−55^ The obtained samples present a δ*T* of 0.5 K at *T*_0_, in close correspondence with the primary luminescent thermometers reported for Yb^3+^/Er^3+^-containing materials (see Table 2 in ref (32)).

It is important to stress that, despite both PY14 and PY18 samples presenting Yb^3+^ and Pr^3+^ in their composition, the energy levels of Yb^3+^ do not participate in the thermalization process between the ^3^P_1_ and ^3^P_0_ thermally coupled levels of Pr^3+^, and thus they do not affect the thermometric performance of Pr^3+^. Hence, once the thermometric response of these Pr^3+^/Yb^3+^ co-doped fluoride phosphate glasses is solely due to light emission arising from Pr^3+^, we may conclude that these samples can operate as luminescent primary temperature sensors in different media as far as we guarantee (i) the structural integrity of the vitreous matrix and (ii) no overlapping is occurring between the Pr^3+^ emission and the absorption of an eventual light emitting material placed in the optical path of the samples. These findings indicate that embedding Pr^3+^ into a vitreous matrix is an ingenious strategy for developing highly accurate luminescent thermometers because it does not suffer from temperature deviations from laser-induced heating observed in power-dependent approaches, reducing thermal and spectral artifacts, besides operating in a wide temperature range without requiring time-consuming calibration curves.

## 4 Conclusions

We successfully prepared and characterized luminescent fluoride phosphate glasses co-doped with Pr^3+^ and Yb^3+^ performing the structural, thermal, optical, and thermometric evaluation of the glasses. The NMR results showed significant network modification resulting from the addition of alkaline-earth and yttrium fluorides, which led to the depolymerization of phosphate chains through the breaking of P–O–P linkages. A clear preference for Li-phosphate over Li-fluoride interaction was observed and the formation of P–F bonds is largely suppressed in the obtained samples. While the incorporation of Yb^3+^ can induce crystallization, the DSC results suggest that co-doping the samples does not substantially alter the connectivity of the fluoride phosphate glass network. The temperature-dependent emission spectra of the co-doped samples demonstrated the inherent ability of Pr^3+^ to work as a primary thermometer irrespective of the doping content, making these samples suitable for real-world applications due to their elevated thermal stability.

Overall, our work represents a significant contribution to the field of luminescence thermometry by proposing a simpler, faster, and more reliable approach for temperature readouts. The obtained Pr^3+^/Yb^3+^ co-doped fluoride phosphate glasses constitute a promising platform for the development of cost-effective, accurate, and high-performance temperature sensors, particularly in applications such as biomedical sensors and wearable technology, where a vitreous matrix that can be molded into different shapes is required. These findings demonstrate the potential use of Pr^3+^-containing materials as luminescent thermometers that do not require thermal calibration.

## Supporting Information Available

Detailed preparation and characterization procedures, advanced NMR studies and molecular dynamics simulations, and additional details of the thermometric performance (PDF)
