# Supplementary material for: Extending the Palette of Luminescent Primary Thermometers: Yb3+/Pr3+ Co-Doped Fluoride Phosphate Glasses
Source: Chem Mater. 2023 Aug 2;35(17):7229–38. doi: 10.1021/acs.chemmater.3c01508 (PMC10500981; doi:10.1021/acs.chemmater.3c01508)
Supplement: Supplementary file 1 — cm3c01508_si_001.pdf [file cm3c01508_si_001.pdf]

SUPPORTING INFORMATION

Extending the palette of luminescent primary thermometers: Yb^{3+}/Pr^{3+} co-doped fluoride phosphate glasses

Fernando E. Maturi[1,2], Anuraag Gaddam[3], Carlos D. S. Brites[1], Joacilia M. M. Souza[4], Hellmut Eckert[3], Sidney J. L. Ribeiro[2], Luís D. Carlos[1], Danilo Manzani[4*]*

[1]Phantom-g, CICECO - Aveiro Institute of Materials, Department of Physics, University of Aveiro, 3810-193 Aveiro, Portugal
[2]Institute of Chemistry, São Paulo State University (UNESP), 14800-060 Araraquara, SP, Brazil
[3]São Carlos Institute of Physics, University of São Paulo, IFSC-USP, 13566-590 São Carlos, SP, Brazil
[4]São Carlos Institute of Chemistry, University of São Paulo, IQSC-USP, 13560-970 São Carlos, SP, Brazil

*Corresponding authors: lcarlos@ua.pt and dmanzani@usp.br

Table of Contents

S1. Supplementary text 2

S1.1. Preparation and characterization of the fluoride phosphate glasses 2

S1.2. Solid-state NMR structural studies 2

S1.2.1 Molecular dynamics simulations 5

S1.3. Energy gap determination of thermally-coupled levels of Pr^{3+} 5

S1.4. Relative thermal sensitivity and uncertainty in temperature 6

S2. Supplementary figures 7

S3. Supplementary tables 18

S4. References 19

S1. Supplementary text

S1.1. Preparation and characterization of the fluoride phosphate glasses

Fluoride phosphate glasses were prepared in batches of 5 g by weighing the raw materials described in **Table S1**, followed by their thorough homogenization in an agate mortar, loading the resulting powder mixture into a platinum crucible, which was covered for melting. The glass samples were obtained by melting the precursors at 1373 K for 30 min to ensure homogenization, cast into a stainless-steel mold pre-heated at 563 K, and annealed at the same temperature for 3 h before cooling it slowly to room temperature. The bulk samples were cut into equal pieces of 1 cm^2 and optically polished with parallel faces and the same thickness (2 mm) for further optical characterizations. The characteristic temperatures, relative absorption strengths, and absolute emission quantum yields of the obtained fluoride phosphate glasses are summarized in **Table S2**, **Table S3**, and **Table S4**, respectively.

S1.2. Solid-state NMR structural studies

The one-dimensional (1D) ^{31}P experiments were recorded using single-pulse acquisition with a $\pi/2$ pulse length of 3.05 μs. Relaxation delays of 900, 10, and 5 s were used for samples PY00, PY14, and PY18, respectively. The ^{31}P chemical shifts were referenced with respect to BPO_4 at −29.3 ppm *versus* 85% H_3PO_4. Two-dimensional ^{31}P J-resolved NMR spectra were measured in a 3.2 mm probe spinning at 10.0 kHz, using π and $\pi/2$ pulses of 3.0 μs and 6.0 μs length, and a relaxation delay of 80 s, following a pre-saturation pulse train. Rotor synchronized echoes were recorded up to an evolution time of 31 ms. Data were acquired using a 32-step phase cycle. ^{31}P refocused (R-) incredible natural abundance double quantum transfer experiment (INADEQUATE) experiments[1] were conducted on a 600 MHz NMR spectrometer (Avance Neo 600, Bruker) operating at 14.1 T, using a 2.5 mm probe with MAS rate of 15.0 kHz. The $\pi/2$ pulse length and recycle delay were 1.825 μs and 60 s, respectively. A single mixing time (2τ) of 8.33 ms, corresponding to a ^{31}P–^{31}P J-coupling constant of 30.0 Hz, was employed. Under the same conditions (pulse length, recycle delay, MAS rate, etc.), a 1D spectrum was recorded for comparison.

The ^{19}F 1D MAS-NMR experiments were recorded using a rotor-synchronized Hahn echo sequence (2 rotor cycles) with a $\pi/2$ pulse length of 3.8 μs. The relaxation delays were 60 s (PY00) and 1 s (PY14 and PY18). The ^{19}F chemical shifts were referenced with respect to NaF at −224

ppm *versus* $CFCl_3$.[2] For $^{31}P\{^{19}F\}$ REDOR, the π/2 and π pulse lengths were 3.05 and 6.10 μs, respectively. The dipolar recoupling π pulse lengths on ^{19}F were 7.90 μs, as optimized on Na_2PO_3F. Before the π/2 excitation pulse on ^{31}P, a saturation pulse train was applied to establish reproducible stationary magnetization conditions, and relaxation delays of 5 s (PY18), 60 s (Na_2PO_3F), and 120 s (PY00) were used. ^7Li MAS-NMR spectra were recorded with π/8 pulses of 0.70 μs length as determined using a LiCl solution (1.00 mol L^{-1}). The relaxation delays were 30 s (PY00), 3 s (PY14), and 2 s (PY18). The ^7Li chemical shifts were referenced with respect to a LiCl solution (1.00 mol L^{-1}) at 0 ppm.

Since these glasses are prone to F volatilization, the amount of F loss was quantified using a ^{19}F NMR Hahn-Echo sequence. Known quantities of the samples and NaF were ground together and packed in a rotor. To correct potential errors due to different spin-spin relaxation times, the spectra were measured with 2, 4, 6, and 8 rotor cycles. The ratios of the signal intensities were back-extrapolated to 0 rotor cycles. From this ratio, the amount of F in each sample was calculated. The ^6Li MAS-NMR spectra were recorded using a Hahn echo sequence with a π/2 and π pulse lengths of 6.2 and 12.4 μs (optimized on a glassy $LiPO_3$ sample), using a 3.2 mm XY probe, operated at a spinning speed of 15.0 kHz. Relaxation delays were 60, 6, and 6 s, for samples PY00, PY14, and PY18, respectively. The ^6Li chemical shifts were referenced to the LiCl solution (1.00 mol L^{-1}) at 0 ppm. All spectra were analyzed using ssNake[3] and/or SIMPSON[4] software.

The $^6Li\{^{31}P\}$ REDOR measurements on the undoped sample were done on the same probe, using the Schaefer-Gullion sequence.[5] The pulse lengths were optimized using glassy $LiPO_3$. The π/2 and π pulse lengths on ^6Li were 6.2 and 12.4 μs, respectively. The dipolar recoupling pulse lengths on ^{31}P were 13.0 μs. Before the π/2 excitation pulse on ^6Li, a saturation pulse train was applied, and the REDOR experiments were conducted with relaxation delays of 60 s (PY00). Following previously published procedures, the data were analyzed in terms of the approximate equation:[6]

$$\frac{\Delta S}{S_0} = \frac{S_0 - S}{S_0} = f\frac{4}{3\pi^2}M_{2(Li-P)}(n\tau_r)^2 \qquad \textbf{(S1)}$$

to yield a dipolar second moment $M_{2(Li–P)}$ characterizing the average square of the strength of the magnetic dipole-dipole coupling between the observed nucleus ^6Li and the heteronuclear ^{31}P, with $\Delta S/S_0$ corresponding to the normalized signal intensity in the presence (intensity S) and the absence (intensity S_0) of the recoupling pulses. This approximate expression holds in the limit of short

mixing times, where $\Delta S/S_0 \leq 0.2$. Here $n\tau_r$, the number of rotor cycles times the rotor period, defines the dipolar mixing time applied in the experiment and f is a scaling factor obtained by comparing the experimental M_2 value with that of a crystalline reference compound (which can be calculated from the internuclear distances in the crystal structure). It is important that the reference compound presents similar spin dynamics as the glasses and that the data on the glasses and reference compound are measured under identical conditions. As in the present case, no reference compound was available, only the raw data obtained on the two glasses were compared (*i.e.*, we assumed $f = 1$). Static ^{31}P spin echo decay data were measured on an NMR spectrometer system (Avance III Neo, Bruker) operating at a magnetic field strength of 9.4 T, using the Hahn echo sequence $\pi/2$-t_1-π-t_1. The $\pi/2$ and π pulse lengths were 8.3 and 16.6 ms, and a relaxation delay of 300 s was used. The data were analyzed in terms of a Gaussian decay:[7]

$$\frac{I}{I_0} = \exp\left\{ -\frac{1}{2}M_{2(P-P)}(2t_1)^2 \right\} \qquad \textbf{(S2)}$$

yielding the dipolar second moment $M_{2(P-P)}$ as a measure of the average squared strength of the internuclear magnetic dipole-dipole coupling. For a semi-quantitative interpretation of the second moment values $M_{2(Li-P)}$ and $M_{2(P-P)}$, Monte Carlo calculations of random atomic arrangements in space were conducted by randomly generating Li, P, and F atoms in a cubic box, with interatomic cutoff distances based on crystalline materials at concentrations based on density measurements. The periodic boundary conditions were assumed throughout. Then the $M_{2(A-B)}$ values were calculated using:

$$M_{2(A-B)} = \frac{4}{15}\left(\frac{\mu_0}{4\pi}\right)^2 \gamma_A^2\gamma_B^2\hbar^2 S(S+1)\frac{1}{N_A}\sum_{i=1}^{N_A}\sum_{j=1}^{N_B}\frac{1}{r_{ij}^6} \qquad \textbf{(S3)}$$

The prefactor of 4/15 was assumed for both homo- and heteronuclear cases, as the spin-exchange ("flip-flop") term of the homonuclear dipolar Hamiltonian can be considered quenched in glasses where a wide dispersion of resonance frequencies leads to a low probability that nuclei in the proximity of each other have identical resonance frequencies. The parameters γ, $\hbar$, S, r_{ij}, N_A, N_B are gyromagnetic ratios, Dirac constant, nuclear spin quantum number of the nuclei the observed spins are interacting with, internuclear distances, number of observed and unobserved nuclei, respectively. In addition, molecular dynamics (MD) simulations of $LiPO_3$ glass were

carried out, using the LAMMPs code[8] with the potentials developed by Pedone *et al*.[9] The exact details and the parameters used to perform these simulations are presented in the following section.

S1.2.1 Molecular dynamics simulations

Molecular dynamics (MD) simulations were carried out using Pedone Potentials.[9] The potential parameters bond dissociation energy (D_{ij}), the function of the slope of the potential energy well (a_{ij}), and equilibrium bond distance (r_0) for Li–O, P–O, and O–O atomic pair interactions are displayed in **Table S5**. To evaluate long-range Coulomb interactions, we utilized the Ewald sum method with a strength precision of 10–5 and a 12 Å cutoff. Our simulation employed the Verlet velocity algorithm to integrate Newton's equations of motion with a simulation time interval of 1 fs. We controlled the temperature and pressure using a Nosé-Hoover thermostat and a barostat every 100 steps. Additionally, we employed periodic boundary conditions in all three dimensions throughout the simulation, which involved 10,000 atoms initially distributed randomly in a cubic simulation box with dimensions corresponding to the experimental density of the $LiPO_3$ glass (2.34 g cm^{-3}). After minimizing the energy, we heated the glasses from 300 to 5000 K at a rate of 100 K ps^{-1} using the canonical NVT ensemble (Evans thermostat). The sample was held at the melting temperature of 5000 K for 100 ps under the NVT ensemble, then cooled to 300 K at rates of 5 K ps^{-1} under NVT conditions and annealed at 300 K for 100 ps under NVT and 100 ps under NPT. Finally, we analyzed the obtained structures using a laboratory-developed code to calculate the pair distribution functions, the second dipole-dipole moments, and spin echo decay (SED) curves.

S1.3. Energy gap determination of thermally-coupled levels of Pr^{3+}

The areas, peak energies, and widths of the emission bands corresponding to the $^3P_1 \rightarrow {}^3H_5$ and $^3P_0 \rightarrow {}^3H_5$ transitions of Pr^{3+} were calculated from the deconvolution of the emission spectra of the glass samples under 443 nm excitation at room temperature by using a custom script written in MATLAB 2022a under the license provided to the University of Aveiro. In the first step, a polynomial baseline correction was performed to remove the electric noise from the spectrofluorometer signal, followed by the conversion of the emission spectrum from wavelength (nm) to energy (cm^{-1}) units by applying the Jacobian conversion.[10,11] The second step consisted of adjusting 5 Gaussian functions to the energy-converted emission spectra between the 17700–

19600 cm^{-1} spectral range, which is the minimum number of Gaussian functions required to get a good fit ($r^2 > 0.998$ for both samples). In the final step, the barycenters of $^3P_1 \rightarrow {}^3H_5$ and $^3P_0 \rightarrow {}^3H_5$ transitions of Pr^{3+} were computed as their weighted arithmetic means by using the areas and peak energies retrieved from the abovementioned spectral Gaussian deconvolution using the emission spectra of the samples measured at room temperature. The value of ΔE corresponds to the difference between the barycenters of the emission bands assigned to the $^3P_1 \rightarrow {}^3H_5$ (I_2, two Gaussian functions) and $^3P_0 \rightarrow {}^3H_5$ (I_1, three Gaussian functions) transitions of Pr^{3+}, as shown in **Figure S1**. The uncertainty in ΔE ($\delta\Delta E$) was calculated by propagating the uncertainty in ΔE, taking into account the uncertainties of the areas and peak energies of each Gaussian component.

S1.4. Relative thermal sensitivity and uncertainty in temperature

The absolute sensitivity $\left(S_a = \frac{\partial \Delta}{\partial T}\right)$ indicates how much a given thermometric parameter Δ changes by increasing one unit of temperature. Although S_a is a good measure of the temperature dependence of Δ, S_a values of different materials cannot be compared because it is an intrinsic response of the temperature-dependent luminescence of each material. Therefore, the relative thermal sensitivity $\left(S_r = \frac{1}{\Delta}\left|\frac{\partial \Delta}{\partial T}\right| = \frac{|S_a|}{\Delta}\right)$ was introduced as a figure of merit to perform quantitative comparisons between different materials, which is the absolute value of S_a normalized by the magnitude of Δ, usually presented in units of percentage change per unit of temperature change (% K^{-1} or % °C^{-1}).

Once the thermometric parameter Δ describing the emission arising from the thermally coupled levels of Pr^{3+} is given by the Boltzmann distribution (Equation 1 of the manuscript), S_r assumes the following form for a Boltzmann-based primary thermometer:

$$S_r = \frac{\left|\frac{d}{dT}\left(B\exp\left(-\frac{\Delta E}{k_B T}\right)\right)\right|}{B\exp\left(-\frac{\Delta E}{k_B T}\right)} = \frac{\left|\frac{\Delta E}{k_B T^2}B\exp\left(-\frac{\Delta E}{k_B T}\right)\right|}{B\exp\left(-\frac{\Delta E}{k_B T}\right)} = \frac{\Delta E}{k_B T^2} \qquad \textbf{(S1)}$$

where S_r depends on the energy gap ΔE between the $^3P_1$ and $^3P_0$ emitting levels of Pr^{3+} and the temperature T of the medium. Once ΔE is nearly constant in the temperature range studied in this work, S_r gives higher values for lower temperatures. Nonetheless, we herein report the values of S_r at room temperature (T_0) to make it more suitable for comparisons to the values previously reported in the literature.

The uncertainty in temperature $\left(\delta T = \frac{1}{S_r}\frac{\delta\Delta}{\Delta}\right)$ is another figure of merit used to assess the reliability of the temperature accuracy of luminescent thermometers. Here, δT represents the smallest temperature that can be resolved by the luminescent thermometer while $\delta\Delta/\Delta$ corresponds to the relative uncertainty of the thermometric parameter Δ, which is given by:

$$\frac{\delta\Delta}{\Delta} = \sqrt{\left(\frac{\delta I_2}{I_2}\right)^2 + \left(\frac{\delta I_1}{I_1}\right)^2} \tag{S2}$$

where δI_2 and δI_1 are the uncertainties in the integrated intensities I_2 and I_1, respectively, estimated by dividing the readout fluctuations of the baseline by the maximum intensity value. Both samples presented a $\delta\Delta/\Delta$ of 0.49%, corresponding to a signal-to-noise ratio (SNR) of 204. **Table S6** displays the parameters used to perform primary thermal sensing based on the downshifting emission of Pr^{3+} and their corresponding values of S_r and δT.

S2. Supplementary figures

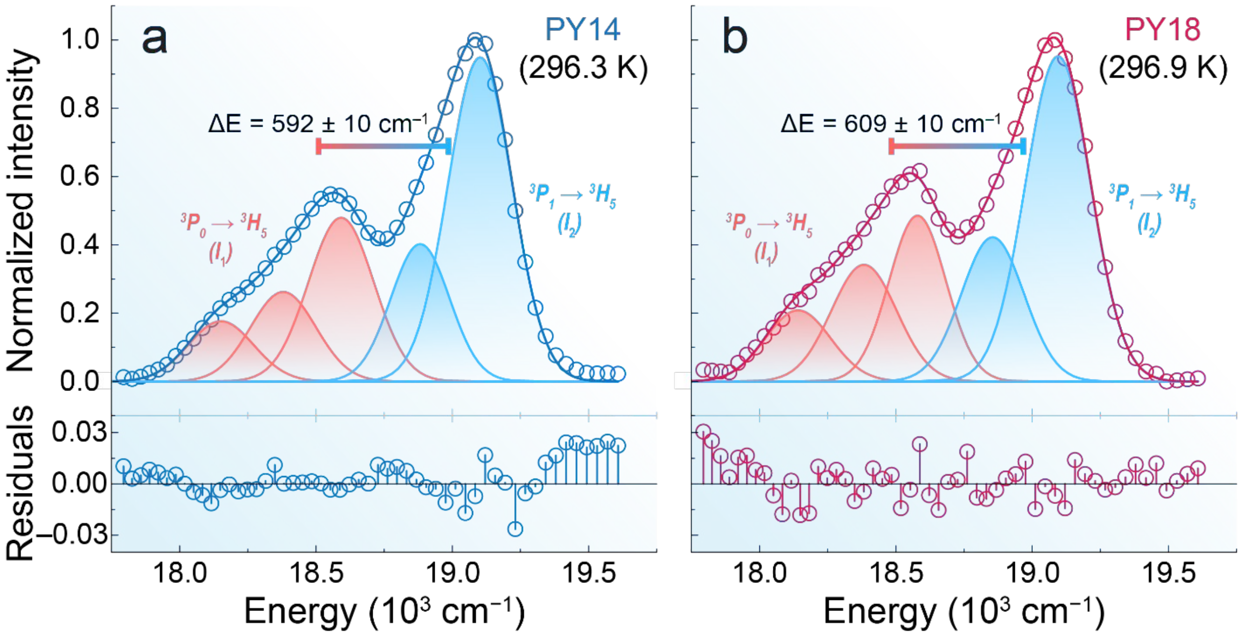

Figure S1. Spectral deconvolution of the emission spectra of samples **(a)** PY14 and **(b)** PY18 measured under excitation at 443 nm. The symbols and the solid darker lines are the emission spectra and the fit envelope, respectively. The Gaussian functions in light red and light blue arise from the $^3P_0 \rightarrow {}^3H_5$ (I_1, three components) and $^3P_1 \rightarrow {}^3H_5$ (I_2, two components) transitions of Pr^{3+}, respectively.

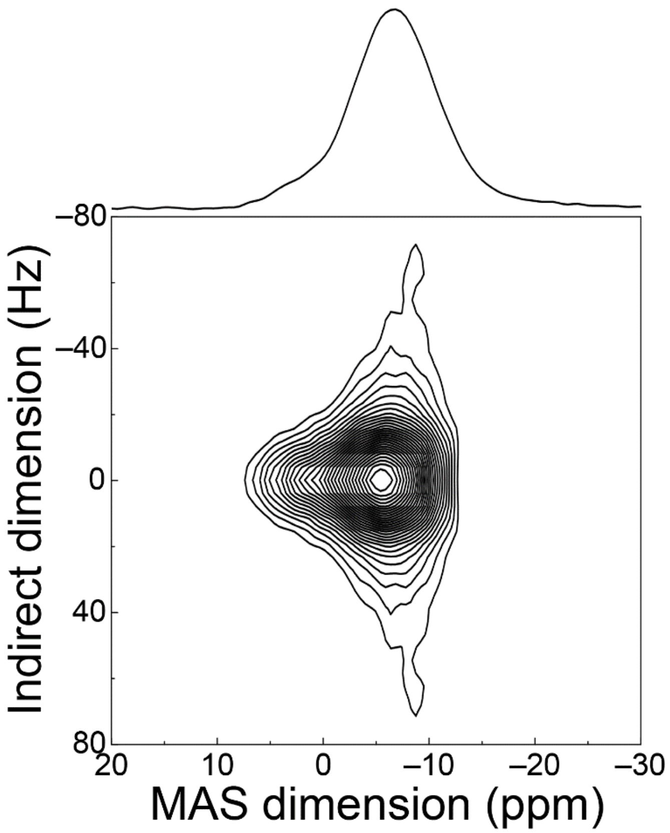

Figure S2. ^{31}P 2D J-resolved MAS-NMR spectrum of the PY00 sample. The spectrum projected on the top part of the figure was taken at an indirect dimension of 0 Hz.

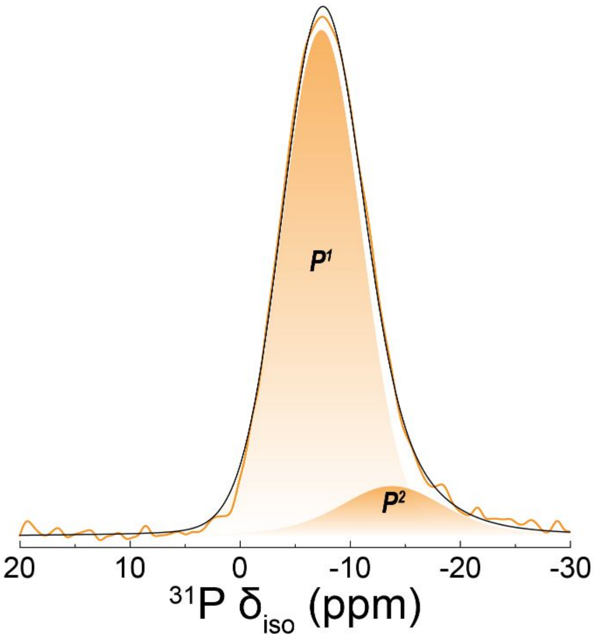

Figure S3. ^{31}P R-INADEQUATE data of the PY00 sample. The solid orange line is the obtained signal, the shadowed areas correspond to the phosphate units, and the dashed black line is the fitted curve.

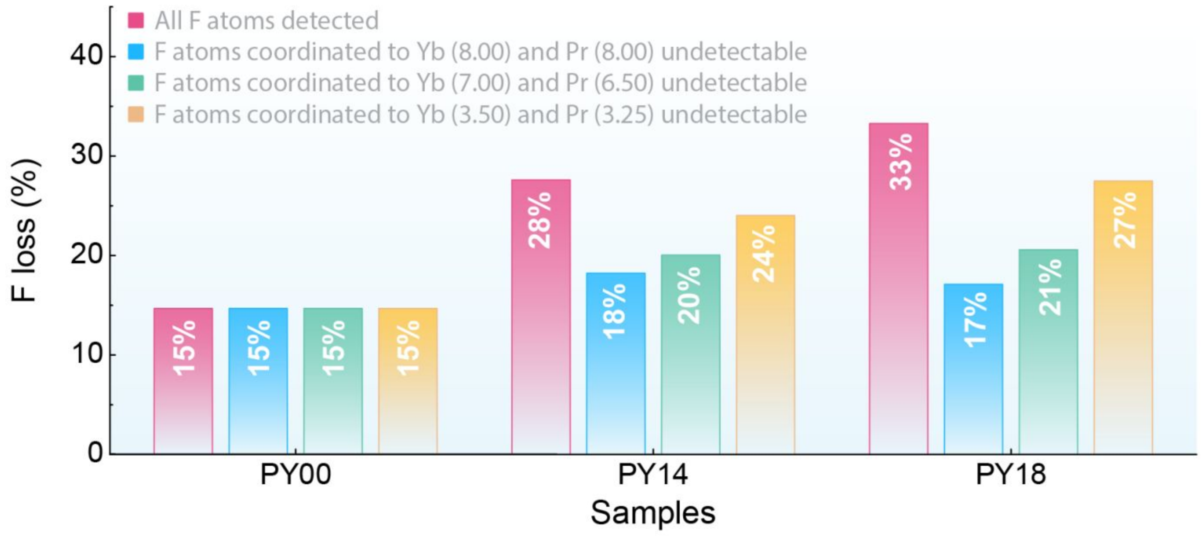

Figure S4. Fluorine (F) loss estimated by ^{19}F MAS-NMR. The results shown in pink are the uncorrected loss values where all F atoms are detected. The results in blue, green, and yellow display the corrected loss values when considering that only a few F atoms are detected due to the coordination to Pr^{3+}/Yb^{3+} with coordination numbers of 8.00/8.00 in crystalline fluoride, 7.00/6.50 in vitreous fluoride-only, and 3.50/3.25 in mixed vitreous fluoride phosphate environments, respectively. The mean corrected F loss values are presented in Figure 2b of the manuscript, taking into account the data from these three distinct coordination scenarios.

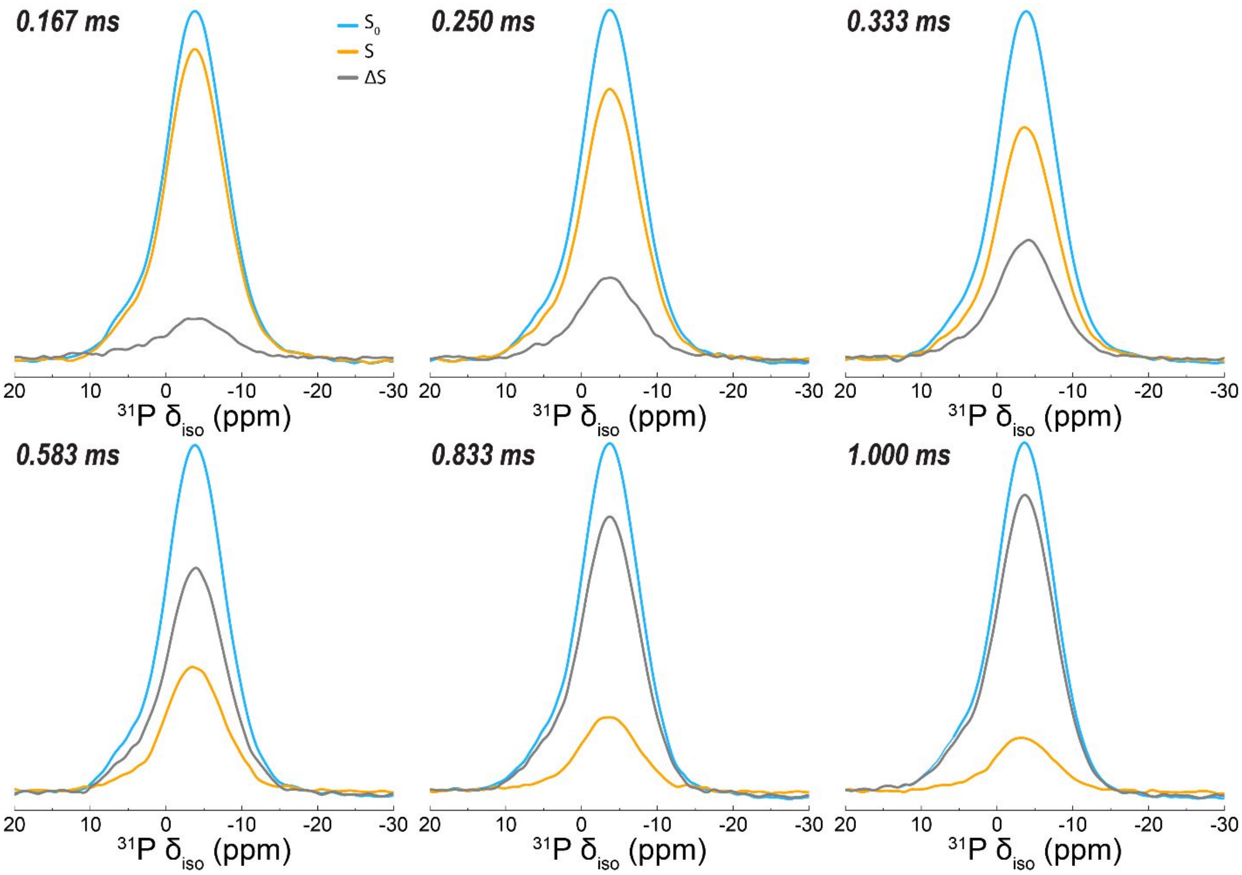

Figure S5. Fourier transforms of the $^{31}P\{^{19}F\}$ REDOR data (yellow curves) and the corresponding REDOR difference signal ($\Delta S = S_0 - S$, gray curves) for a dipolar mixing time between 0.167 and 1.000 ms. The reference signal S_0 is shown in blue. The chemical shifts of both signals are identical within the experimental uncertainty, arguing against a significant contribution from the F-bonded P species.

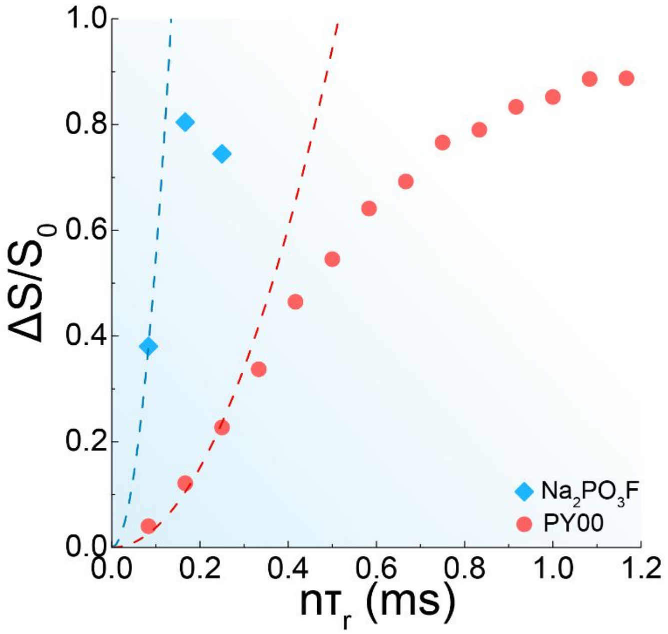

Figure S6. $^{31}P\{^{19}F\}$ REDOR dephasing curves displaying the normalized signal intensity as a function of the dephasing time $n\tau_r$ for the sample PY00 and the model compound Na_2PO_3F. The solid symbols are the experimental results while the dashed lines are the parabolic approximation in the initial regime of the obtained data ($\Delta S/S_0 \leq 0.2$) given by **Equation S1**, resulting in an uncorrected value of $M_{2(P\text{–}F)}$ of 28 $\text{Mrad}^2\ \text{s}^{-2}$. Measurements were carried out at 5.7 T and a spinning frequency of 24.0 kHz.

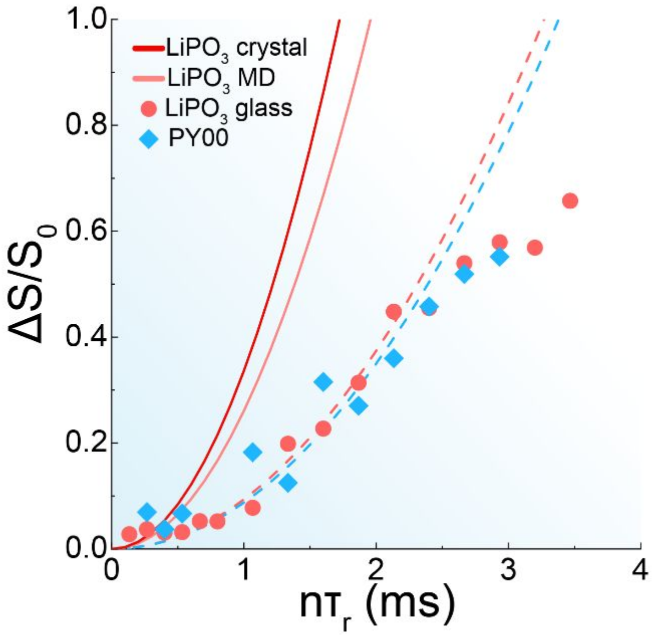

Figure S7. $^{6}Li\{^{31}P\}$ REDOR curves measured for PY00 (diamonds) and $LiPO_3$ glass (circles). Dashed curves indicate fits of the data to Equation S1, while solid curves indicate the expected REDOR behavior for crystalline $LiPO_3$ (dipolar coupling based on the internuclear distances in the crystal structure) and an MD simulation output for $LiPO_3$ glass. The deviation of the experimental data of $LiPO_3$ glass and the parabola calculated from the MD output suggests a calibration factor of 0.33.

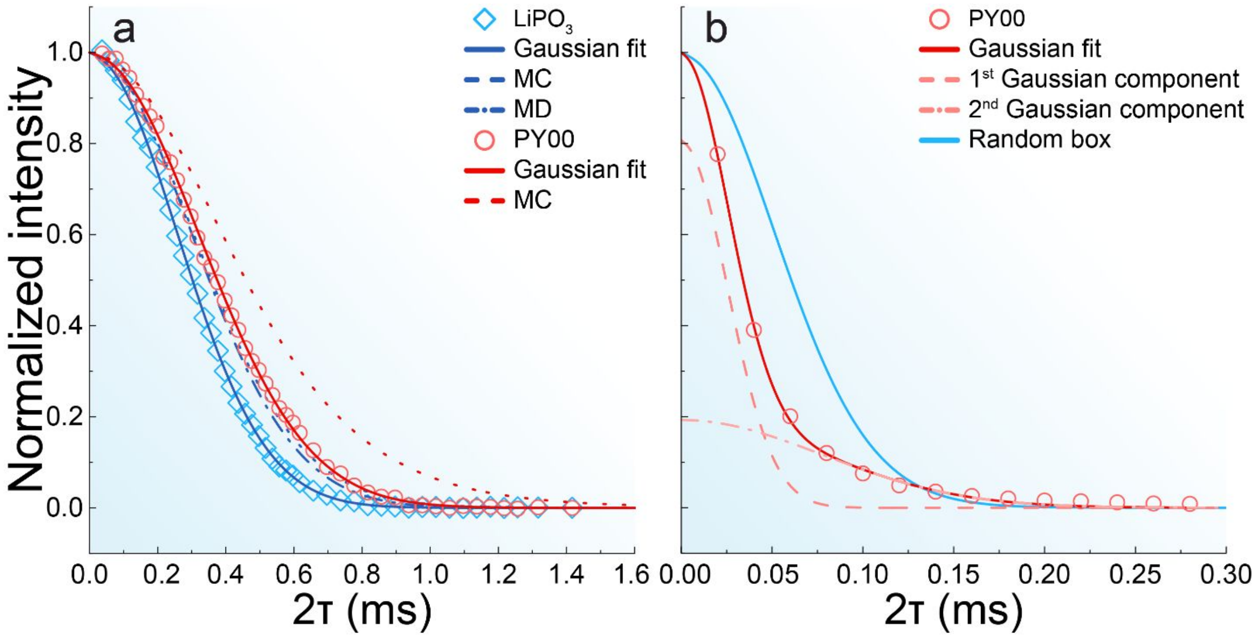

Figure S8. (a) Static ^{31}P SED curves of glasses PY00 (red) and $LiPO_3$ (blue). Solid curves show the fits to **Equation S2**, yielding $M_{2(P-P)}$ values of 9.9 Mrad^2 s^{-2} and 15.2 Mrad^2 s^{-2}, respectively. The dashed and dotted lines are simulated SED curves from MD and Monte Carlo (MC) simulations. **(b)** Static ^{19}F SED curves of the sample PY00.

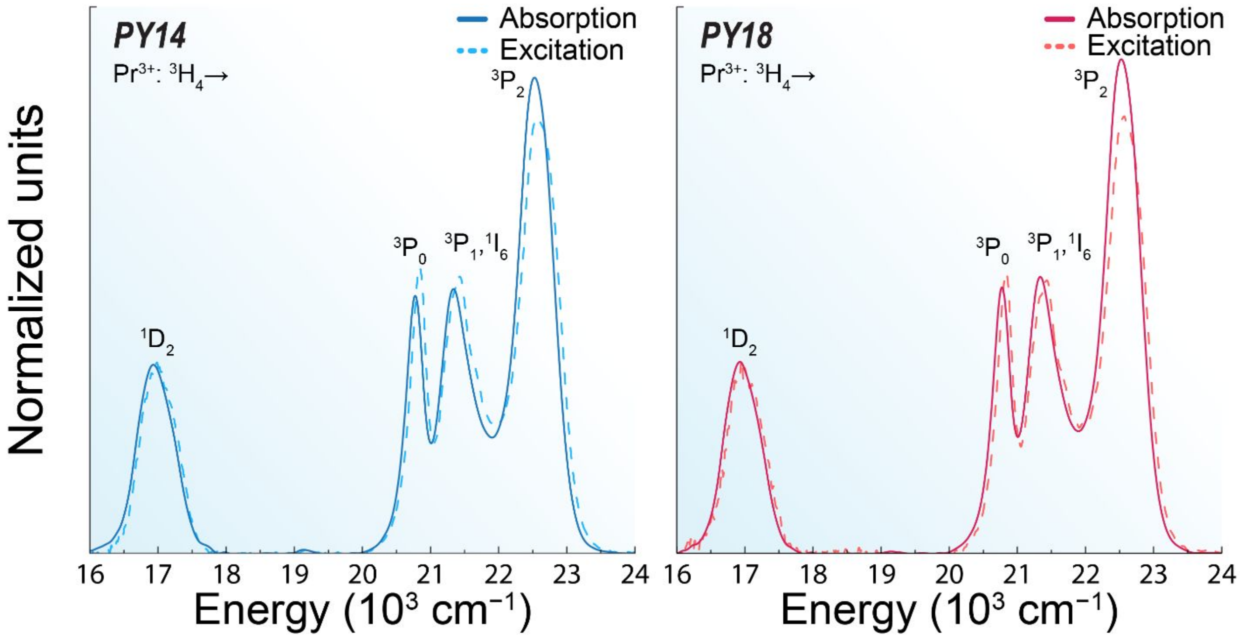

Figure S9. Absorption (solid curves) and excitation (dashed curves) spectra for the PY14 and PY18 samples measured at room temperature. The excitation spectra were recorded by monitoring the emission of Yb^{3+} at 979 nm. All the data were converted from wavelength to energy units by using the Jacobian conversion and then normalized at the $^3H_4 \rightarrow {}^1D_2$ peak for comparison purposes.

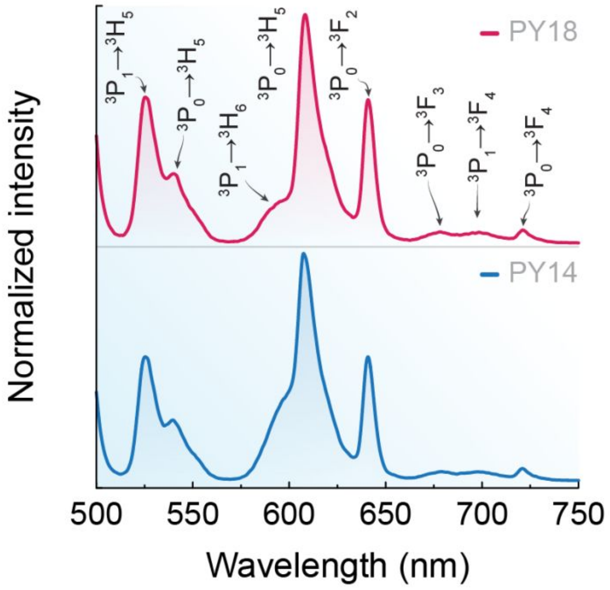

Figure S10. Upconversion emission spectra of the samples PY14 (bottom) and PY18 (top) spectra for the PY14 and PY18 samples measured at room temperature under continuous wave laser excitation at 980 nm (150 W cm^{-2}).

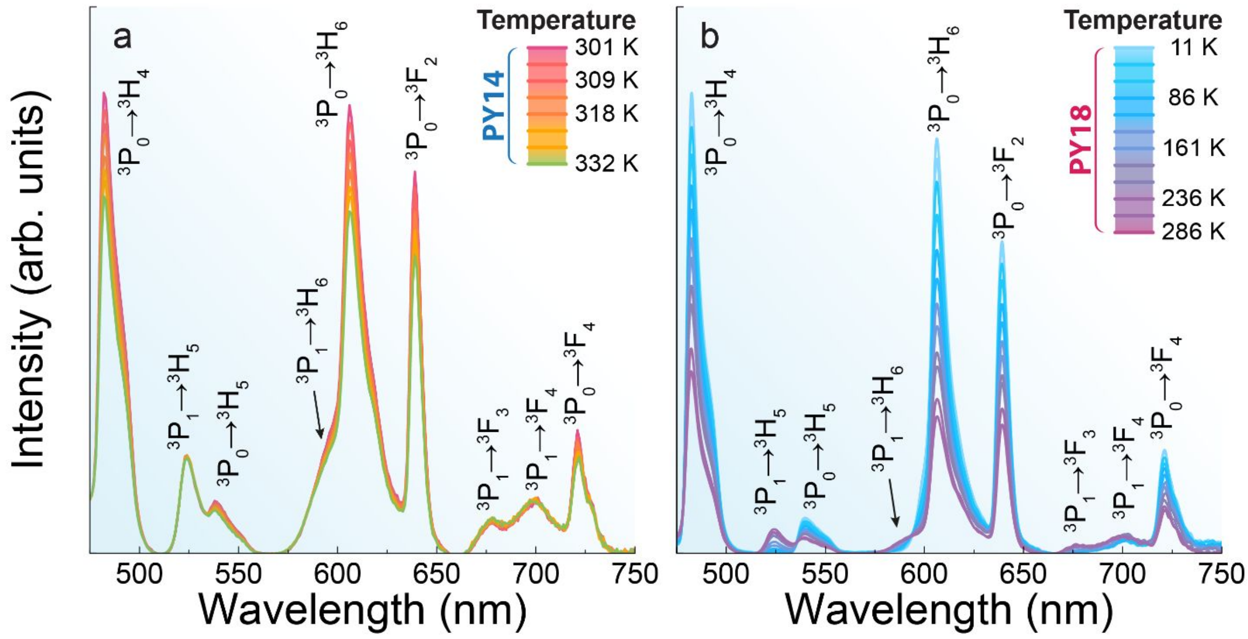

Figure S11. Temperature-dependent emission spectra of the samples **(a)** PY14 and **(b)** PY18 under excitation at 443 nm. The *y*-axis was rescaled for better visualization of all the curves in each panel.

S3. Supplementary tables

Table S1. Chemical compositions and densities of the prepared fluoride phosphate glasses.

Sample	Chemical composition (mol%)						Density
	$LiPO_3$	YF_3	SrF_2	CaF_2	Pr_7O_{11}	Yb_2O_3	(g cm^{-3})
PY00	50.000	20.000	20.000	10.000	-	-	3.48
PY14	49.375	19.750	19.750	9.875	0.250	1.000	3.62
PY18	48.875	19.550	19.550	9.775	0.250	2.000	3.74

Table S2. Characteristic temperatures of the obtained glass samples.

Sample	T_g (± 2 K)	T_x (± 2 K)	ΔT (± 4 K)
PY00	602	781	179
PY14	604	741	137
PY18	602	736	134

Table S3. Integrated areas of the absorption and excitation spectra (in arbitrary units) measured at room temperature of the fluoride phosphate co-doped glass samples. The relative absorption strengths (unitless) are given by the ratio between the $^3H_4 \rightarrow {}^3P_{2-0}, {}^1I_6$ and $^3H_4 \rightarrow {}^1D_2$ emission bands.

Pr^{3+} transition	PY14		PY18	
	Absorption	Excitation	Absorption	Excitation
$^3H_4 \rightarrow {}^1D_2$	710.4	681.2	699.2	703.7
$^3H_4 \rightarrow {}^3P_{2-0}, {}^1I_6$	3186.6	3239.0	3329.6	3205.7
Relative strength	4.5	4.8	4.8	4.6

Table S4. Absolute emission quantum yield (q) of Pr^{3+} in the glass samples in the visible spectral range under excitation at 443 nm, measured at room temperature. The values of q for the near-infrared emission of Yb^{3+} were not determined once the light emission was too faint to be detected by the equipment.

Sample	q
PY14	0.026 ± 0.003
PY18	0.018 ± 0.002

Table S5. Parameters of the potentials used in the MD simulations.[9]

Pair	D_{ij} (eV)	a_{ij} (Å^{-2})	r_0 (Å)	C_{ij} (eV Å^{12})
$Li^{0.6}$□$O^{-1.2}$	0.001114	3.429506	2.681360	1.0
$P^{3.0}$□$O^{-1.2}$	0.831326	2.585833	1.800790	1.0
$O^{-1.2}$□$O^{-1.2}$	0.042395	1.379316	3.618701	22.0

Table S6. Energy gap, room temperature, thermometric parameter at T_0 (Δ_0), relative thermal sensitivity, and uncertainty in temperature of samples PY14 and PY18.

Sample	ΔE (cm^{-1})	T_0 (K)	Δ_0	S_r (% K^{-1})	δT (K)
PY14	592 ± 10	296.3 ± 0.1	1.3277	0.97	0.5
PY18	609 ± 10	296.9 ± 0.1	1.2587	0.99	0.5

S4. References

(1) Cadars, S.; Sein, J.; Duma, L.; Lesage, A.; Pham, T. N.; Baltisberger, J. H.; Brown, S. P.; Emsley, L. The Refocused INADEQUATE MAS NMR Experiment in Multiple Spin-Systems: Interpreting Observed Correlation Peaks and Optimising Lineshapes. *J. Magn. Reson.* **2007**, *188* (1), 24–34.

(2) Miller, J. M. Fluorine-19 Magic-Angle Spinning NMR. *Prog. Nucl. Magn. Reson. Spectrosc.* **1996**, *28* (3–4), 255–281.

(3) van Meerten, S. G. J.; Franssen, W. M. J.; Kentgens, A. P. M. SsNake: A Cross-Platform Open-Source NMR Data Processing and Fitting Application. *J. Magn. Reson.* **2019**, *301*, 56–66.

(4) Bak, M.; Rasmussen, J. T.; Nielsen, N. C. SIMPSON: A General Simulation Program for Solid-State NMR Spectroscopy. *J. Magn. Reson.* **2000**, *147* (2), 296–330.

(5) Gullion, T.; Schaefer, J. Rotational-Echo Double-Resonance NMR. *J. Magn. Reson.* **1989**, *81* (1), 196–200.

(6) Pan, Y.; Gullion, T.; Schaefer, J. Determination of C–N Internuclear Distances by Rotational-Echo Double-Resonance NMR of Solids. *J. Magn. Reson.* **1990**, *90* (2), 330–340.

(7) Eckert, H. Structural Characterization of Noncrystalline Solids and Glasses Using Solid State NMR. *Prog. Nucl. Magn. Reson. Spectrosc.* **1992**, *24* (3), 159–293.

(8) Thompson, A. P.; Aktulga, H. M.; Berger, R.; Bolintineanu, D. S.; Brown, W. M.; Crozier, P. S.; in ’t Veld, P. J.; Kohlmeyer, A.; Moore, S. G.; Nguyen, T. D.; Shan, R.; Stevens, M. J.; Tranchida, J.; Trott, C.; Plimpton, S. J. LAMMPS - a Flexible Simulation Tool for Particle-Based Materials Modeling at the Atomic, Meso, and Continuum Scales. *Comput. Phys. Commun.* **2022**, *271*, 108171.

(9) Pedone, A.; Malavasi, G.; Menziani, M. C.; Cormack, A. N.; Segre, U. A New Self-Consistent Empirical Interatomic Potential Model for Oxides, Silicates, and Silica-Based Glasses. *J. Phys. Chem. B* **2006**, *110* (24), 11780–11795.

(10) Mooney, J.; Kambhampati, P. Get the Basics Right: Jacobian Conversion of Wavelength and Energy Scales for Quantitative Analysis of Emission Spectra. *J. Phys. Chem. Lett.* **2013**, *4* (19), 3316–3318.

(11) Mooney, J.; Kambhampati, P. Correction to “Get the Basics Right: Jacobian Conversion of Wavelength and Energy Scales for Quantitative Analysis of Emission Spectra.” *J. Phys. Chem. Lett.* **2014**, *5* (20), 3497–3497.
